# Organization of the Thermal Grill Illusion by Spinal Segments

**DOI:** 10.1002/ana.25307

**Published:** 2018-09-03

**Authors:** Francesca Fardo, Nanna Brix Finnerup, Patrick Haggard

**Affiliations:** ^1^ Institute of Cognitive Neuroscience University College London London United Kingdom; ^2^ Danish Pain Research Center, Department of Clinical Medicine Aarhus University Aarhus Denmark; ^3^ Interacting Minds Center Aarhus University Aarhus Denmark

## Abstract

**Objective:**

A common symptom of neuropathy is the misperception of heat and pain from cold stimuli. Similar cold allodynic sensations can be experimentally induced using the thermal grill illusion (TGI) in humans. It is currently unclear whether this interaction between thermosensory and nociceptive signals depends on spinal or supraspinal integration mechanisms. To address this issue, we developed a noninvasive protocol to assess thermosensory integration across spinal segments.

**Methods:**

We leveraged anatomical knowledge regarding dermatomes and their spinal projections to investigate potential contributions of spinal integration to the TGI. We simultaneously stimulated a pair of skin locations on the arm or lower back using 1 cold (∼20°C) and 1 warm thermode (∼40°C). The 2 thermodes were always separated by a fixed physical distance on the skin, but elicited neural activity across a varying number of spinal segments, depending on which dermatomal boundaries the 2 stimuli spanned.

**Results:**

Participants consistently overestimated the actual cold temperature on the skin during combined cold and warm stimulation, confirming the TGI effect. The TGI was present when cold and warm stimuli were delivered within the same dermatome, or across dermatomes corresponding to adjacent spinal segments. In striking contrast, no TGI effect was found when cold and warm stimuli projected to nonadjacent spinal segments.

**Interpretation:**

These results demonstrate that the strength of the illusion is modulated by the segmental distance between cold and warm afferents. This suggests that both temperature perception and thermal–nociceptive interactions depend upon low‐level convergence mechanisms operating within a single spinal segment and its immediate neighbors. Ann Neurol 2018;84:463–472

The spatial and temporal integration of fluctuations in skin temperature is fundamental for thermal perception, thermoregulatory behavior, and homeostasis. One key feature of the thermosensory system is robust spatial summation over large skin areas,^1^, ^2^ not only from unimodal thermal stimulation, but also when qualitatively different thermal stimuli are simultaneously applied. Interestingly, when innocuous warm and cold stimuli are alternated on the skin, spatial summation generates a sensation that is seemingly unrelated to the constituent temperatures. In this case, participants often report paradoxical heat sensations,^3^, ^4^ coupled with an intense burning pain.^5^, ^6^, ^7^ These paradoxical heat and pain sensations are commonly known as the thermal grill illusion (TGI), and are key features of sensory integration between simultaneous cold and warm signals. Crucially, the neurophysiological mechanisms underlying the TGI are still a matter of debate. In particular, it is currently unclear whether the TGI is spinally^4^, ^6^, ^8^ or supraspinally mediated,^9^, ^10^ or instead depends upon both spinal and thalamocortical interactions.^7^, ^11^ This limits not only our understanding of the mechanisms underlying thermosensation and pain, but also potential applications of TGI to investigate disruption in clinical disorders such as neuropathy.

One previous study investigating the spatial boundaries of the TGI found that cold and warm spatial summation evoked similar TGI sensations within and across dermatomes.^9^ In the across‐dermatomes condition, cold and warm stimuli are processed by distinct spinal segments, but still evoke TGI sensations. As spinal segments are often considered independent functional units of thermosensory processing, this result has been interpreted as indicating a lack of spinal organization of the TGI. However, this view is an oversimplification of spinal neuroanatomy, as adjacent spinal segments manifest a degree of interconnection. Thinly myelinated A‐delta and unmyelinated C‐fibers, responding to innocuous cold and warm, form synapses not only in the segment corresponding to the root entrance, but also in the nearest 1 to 2 segments via short‐range intersegmental connections known as Lissauer tract.^12^, ^13^

Based on this neuroanatomical argument, we reasoned that thermosensory integration between adjacent spinal segments is not sufficient evidence to conclude that the spinal cord has no integration role in the TGI. Instead, to address the TGI spinal integration hypothesis, we developed a noninvasive behavioral protocol that takes into account the segmental distance between cold and warm spinal afferents. We used 1 cold and 1 warm thermode at fixed distances on the skin, but stimulated cold and warm afferents at distinct segmental distances by varying the locations of 2 thermodes within the same dermatome or across dermatomal boundaries corresponding to adjacent or nonadjacent spinal segments. This procedure enabled us to quantify the degree of integration between cold and warm signals depending on their spinal adjacency. A cardinal feature of the TGI is a paradoxical overestimation of the cold temperature and the associated sensation of burning pain.^7^ We thus hypothesized that this TGI temperature overestimation effect would be modulated by the number of spinal segments between cold and warm afferents. In other words, we expected a reduction of the TGI with increased segmental distance, due to the reduced spinal integration of the underlying thermal signals. A preliminary report of the results was given at the Scandinavian Association for the Study of Pain meeting.^14^

## Subjects and Methods

### *Participants*

A total of 64 healthy volunteers took part in either Experiment 1 (n = 16, 9 females, age = 26.7 ± 4.7 years), Experiment 2 (n = 16, 11 females, age = 23.4 ± 3.7 years), Experiment 3 (n = 16, 8 females, age = 23.6 ± 4.9 years), or Experiment 4 (n = 16, 10 females, age = 23.7 ± 3.9 years) at University College London. All participants were right‐handed by self‐report. Exclusion criteria were history of neurological or psychiatric disorders, sensitive skin on the arms or back, any skin‐related disorder (eg, eczema), and reports of analgesic medication (ie, paracetamol, aspirin, ibuprofen, codeine) or recreational drugs in the 24 hours prior to the experiment. All participants gave written informed consent to participate in the study, and received monetary compensation (£7.50/h) for completing the experiment. Procedures were approved by the University College London research ethics committee, and were carried out in accordance with the guidelines in the Declaration of Helsinki.

### *Procedure*

All experiments took place in a temperature‐controlled room (24°C), and the experimental paradigm included (1) assessment of cold pain, heat pain, and TGI pain thresholds; (2) TGI stimulation; and (3) temperature matching. These procedures were carried out using 3 identical Peltier‐based thermodes, with a 13mm circular diameter (NTE‐2A; Physitemp Instruments, Clifton, NJ). Two thermodes were positioned on the right arm (Experiments 1–3) or right side of the lower back (Experiment 4) for TGI threshold and stimulation. A third thermode was positioned on the left arm for matching the temperature of TGI stimuli in all the experiments. The 3 thermodes were mounted independently, and adjusted at the beginning of each trial, so that the thermal surface was uniformly pressing on the skin.

#### *Pain Thresholds*

Cold pain thresholds (CPTs), heat pain thresholds (HPTs), and thermal grill pain thresholds (TGTs) were measured on 3 different locations on the volar surface of the right forearm (Experiments 1–3) or on the right side of the lower back (Experiment 4). In Experiments 1 and 2, CPT and HPT were measured on the left forearm with 1 thermode, whereas TGT was measured on the right forearm with 2 thermodes. In Experiments 3 and 4, all thresholds were measures using 2 thermodes either on the right forearm (Experiment 3) or on the right side of the lower back (Experiment 4). In CPT and HPT, only 1 thermode decreased or increased in temperature, whereas the second thermode remained at a neutral temperature (30°C). In this way, we ensured that the CPT and HPT measurements were more easily comparable with TGT measurements. The temporal order was fixed: CPT, HPT, and TGT. CPT was always measured first to prevent the preheating effect, which can potentially induce paradoxical heat sensations.^15^ In all 4 experiments, all 3 thresholds were estimated using the method of limits, and each threshold was assessed 3 times, and then averaged. In CPT and HPT, the temperature was decreased or increased at a rate of 0.5°C/s from a baseline temperature of 30°C and participants were instructed to press a button as soon as they felt a painful sensation. In TGT, the temperatures of 2 thermodes were simultaneously decreased (cold thermode) and increased (warm thermode) at a rate of 0.5°C/s from a baseline temperature of 30°C. Specifically, participants were instructed to press a button when they felt either cold or heat pain, from either thermode. The overall temperature range was limited to 5 to 50°C, precluding any risk of skin damage. For each participant, we determined a pair of heat and cold temperatures that were perceived as innocuous in isolation, but which were approximately at TGT when combined. These temperatures were used for the TGI stimulation in the main experimental task.

#### *Thermal Grill Stimulation*

The above thresholding procedure yielded a mean temperature of 19.9 ± 2.3°C (Experiment 1), 20.1 ± 2.1°C (Experiment 2), 19.8 ± 2.6°C (Experiment 3), and 20.0 ± 2.5°C (Experiment 4) for the cold thermode. In contrast, the mean temperatures of the warm thermode were 40.1 ± 2.3°C (Experiment 1), 39.9 ± 2.1°C (Experiment 2), 40.2 ± 2.6°C (Experiment 3), and 40.0 ± 2.5°C (Experiment 4). The thresholding of TGI temperature enabled control of between‐subject variability in thermal sensitivity.^9^ It also ensured that the temperatures did not cause extreme discomfort, but were tolerable throughout the entire duration of each trial.

**Figure 1:**
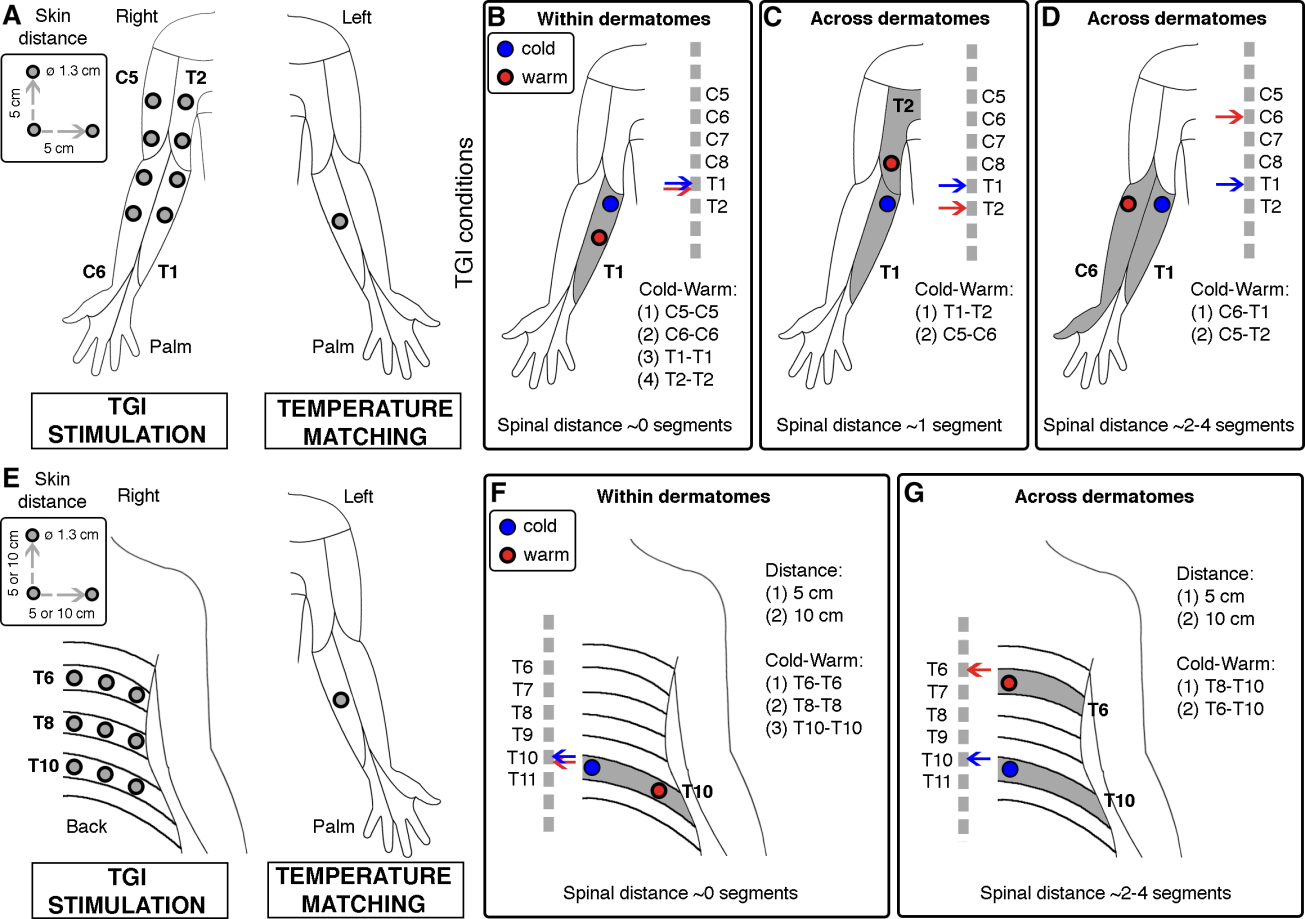
Method. We developed a quantitative approach to measure thermal grill illusion (TGI) perception elicited by cold and warm stimulation delivered by 2 thermodes on the right arm (Experiments 1–3, A–D) or on the right side of the lower back (Experiment 4, E–G). To test whether the TGI is mediated by spinal mechanisms, we varied the configuration of cold and warm stimuli to activate cold and warm afferents at distinct segmental distances. We hypothesized that the strength of the TGI illusion decreased with increased segmental distance between cold and warm afferents. (A) 8 possible locations stimulated with cold and warm thermodes within and across dermatomes on the right arm (TGI stimulation) and approximate location of the matching thermode on the left forearm (TGI matching). The distance between the 2 thermodes was ∼5cm in all conditions. This setup was used in Experiments 1 to 3. (B) Example of within‐dermatome stimulation in Experiments 1 to 3. Cold and warm spinal afferents were most likely within the same spinal segment (ie, ∼0 segments distance). (C) Example of across‐dermatomes stimulation in Experiments 1 and 2. Cold and warm afferents were most likely across 2 adjacent spinal segments (ie, ∼1 segment distance). (D) Example of across‐dermatomes stimulation in Experiments 1 to 3. Cold and warm afferents were most likely across 2 nonadjacent spinal segments (ie, ∼2–4 segments distance). (E) 9 possible locations stimulated with cold and/or warm thermodes within and across dermatomes on the right side of the lower back (TGI stimulation) and approximate location of the matching thermode on the left forearm (TGI matching). The distance between the 2 thermodes was either ∼5 or ∼10cm. This setup was used in Experiment 4. (F) Example of within‐dermatome stimulation in Experiment 4. Cold and/or warm afferents were most likely within the same spinal segment (ie, ∼0 segments distance). (G) Example of across‐dermatomes stimulation in Experiment 4. Cold and warm afferents were most likely across 2 nonadjacent spinal segments (ie, ∼2–4 segments distance). [Color figure can be viewed at http://www.annalsofneurology.org]

In Experiments 1 to 3, TGI stimuli were located within a 2 × 4 grid covering the internal surface of the right forearm and upper arm (Fig 1A). The spacing between adjacent nodes was 5cm in each direction, along the proximodistal and across the mediolateral axis. In Experiment 4, TGI stimuli were located within a 3 × 3 grid on the right side of the lower back (see Fig 1E). The spacing between adjacent nodes was either 5 or 10cm in each direction, along the cephalocaudal and across the mediolateral axes. To test the segmental distance hypothesis, the spatial arrangement of the 2 thermodes was manipulated to deliver the 2 thermal stimuli within the same dermatome or across dermatomal boundaries, based on the American Spinal Injury Association map.^16^, ^17^ Across all experiments, the same stimulation site was never used on consecutive trials, to minimize carryover effects. Furthermore, the relative position of the cold and warm thermodes (cold–warm or warm–cold) was counterbalanced across participants. TGI stimuli were applied for 30 seconds, and then remained in place as the participant began the matching procedure by adjusting the temperature of the contralateral thermode. The duration of each trial was approximately 3 to 5 minutes.

In Experiments 1 and 2, we tested cold–warm pairs within and across dermatomes on the right forearm. In the “within‐dermatome/0 segments” condition, the 2 thermodes were both placed within a single putative dermatome (see Fig 1). Four separate putative dermatomes were tested, by placing the thermodes along the proximodistal axis on the skin innervated by (1) the lateral cutaneous nerve of forearm (most likely, C6), (2) the medial cutaneous nerve of forearm (most likely, C8 or T1), (3) the inferior lateral cutaneous nerve of arm (most likely, C5), or (4) the intercostobrachial nerve of arm (most likely, T2). In the “across‐dermatomes/1 segment” condition, the 2 thermodes were placed along the proximodistal axis between (1) the lateral cutaneous nerve of forearm and the inferior lateral cutaneous nerve of arm (most likely, C5–C6) or (2) the medial cutaneous nerve of forearm and the intercostobrachial nerve of arm (most likely, C8/T1–T2). Finally, in the “across‐dermatomes/2–4 segments” condition, the 2 thermodes were placed along the mediolateral axis between (1) the lateral and the medial cutaneous nerve of forearm (most likely, C6–C8/T1) or (2) the inferior lateral cutaneous and the intercostobrachial nerve of arm (most likely, C5–T2). The thermodes were positioned using standard anatomical landmarks; that is, with respect to the elbow and the midline of the internal surface of the arm. Participants were tested 4 times in each condition, for a total of 12 stimuli. The stimulation order was pseudorandomized, to avoid the stimulation of the same dermatome in 2 consecutive trials.

In Experiments 3 and 4, we tested 4 different combinations of temperatures within‐dermatome (ie, 0 segments) and across‐dermatomes (ie, 2–4 segments). These combinations included 2 types of TGI stimuli (cold–warm, warm–cold), and 2 types of non‐TGI stimuli (cold–cold, warm–warm) to control for unimodal spatial summation. Specifically, in Experiment 3, the thermode locations were identical to the first and third conditions in Experiments 1 and 2 (see Fig 1). Participants were tested 4 times for each stimulation pair and dermatomal condition, for a total of 32 stimuli. In contrast, in Experiment 4, TGI and non‐TGI stimuli were applied on the lower back. We tested locations at 5 and 10cm distance. In the “within‐dermatome/0 segments” condition, the 2 thermodes were both placed within a single putative dermatome, at either 5 or 10cm distance. Three separate putative dermatomes were tested, by placing the thermodes across the mediolateral (ie, transverse plane) on the skin most likely innervated by divisions of the 7th (T7), 8th (T8), 9th (T9), 10th (T10), 11th (T11), or 12th (T12) thoracoabdominal nerves. In the “across‐dermatomes/2–4 segments” condition, the 2 thermodes were placed along the cephalocaudal axis (ie, sagittal plane) most likely between dermatomal areas innervated by the same thoracoabdominal nerves (ie, from T7 to T12). We reasoned that thermodes separated by 5cm would most likely cross 2 dermatomal boundaries, whereas thermodes separated by 10cm would most likely cross 4 dermatomal boundaries. The thermodes were positioned with respect to the waistline and the midline of the back. Participants were tested 3 times for each stimulation pair, dermatomal condition, and skin distance, for a total of 48 stimuli. In both Experiments 3 and 4, the stimulation order was pseudorandomized, to avoid the stimulation of the same dermatome or the application of the same temperature pair in 2 consecutive trials.

#### *Temperature Matching*

After 30 seconds of TGI stimulation, participants were required to estimate the matching temperature using a single thermode on the internal surface of the left arm. In Experiment 1, participants were instructed to match the overall temperature during the TGI stimulation, without focusing particularly on either stimulus location. In contrast, in Experiments 2 to 4, they were instructed to match the temperature of only 1 thermode, which the experimenter indicated by pointing to it. In practice, this was always the cold thermode, but it was never described to participants in this way. In all experiments, participants were instructed to move their left arm to make contact between the internal surface of the left arm and the third thermode, following an auditory cue. This auditory cue was delivered 30 seconds after the beginning of each temperature stimulation. Furthermore, participants were instructed to report whether the matching thermode at baseline temperature (30°C) was cooler or warmer than the sensation generated by the thermode(s) to match. Depending on their initial response, the temperature of the matching thermode was then either decreased or increased at a rate of 0.5°C/s until the participant verbally communicated that the 2 temperatures were highly similar (ie, psychophysical method of adjustment). The experimenter then immediately stopped the temperature change via a button press. In a final phase, the experimenter manually adjusted the temperature in steps of 1, 0.5, 0.2, and 0.1°C, according to the participant's instructions, until an exact temperature match was identified (ie, psychophysical method of limits). The final estimate was therefore independent of response times, rate of temperature change, or stimulus duration on the matching thermode.

Using temperature matching, we investigated whether the combination of cold and warm temperatures (ie, TGI temperature perception) was perceived differently depending on the distance between cold and warm afferents in the spinal cord. The temperature matching procedure was adapted from previous TGI studies.^17^, ^18^, ^19^ Importantly, this procedure avoids response biases that may occur when participants are directly asked to provide pain reports.^7^ Furthermore, we used a different task instruction in Experiment 1 (“match the overall sensation”) relative to Experiments 2 to 4 (“match the sensation from the [cold] thermode”) to control for spatial attention, as spatial integration of afferent signals is either reduced or facilitated depending on whether attention is distributed over 2 stimuli simultaneously, or is alternatively focused on just 1 of 2 stimuli.^20^, ^21^

### *Statistics*

In Experiments 1 and 2, we analyzed the results using 1‐way repeated measures analyses of variance (ANOVAs). We modeled the main effect of segmental distance with 3 levels: (1) 0 segments, (2) 1 segment, (3) 2 to 4 segments. In Experiment 3, we analyzed the results using one 3‐way repeated measures ANOVA. We modeled the interaction between segmental distance (2 levels: 0 vs 2–4 segments), temperature of thermode 1 (2 levels: cold vs warm), and temperature of thermode 2 (2 levels: cold vs warm). Finally, in Experiment 4, we analyzed the results using one 4‐way repeated measures ANOVA. We modeled the interaction between segmental distance (2 levels: 0 vs 2–4 segments), skin distance (2 levels: 5 vs 10 cm), temperature of thermode 1 (2 levels: cold vs warm), and temperature of thermode 2 (2 levels: cold vs warm). Statistical significance was set at *p* < 0.05, and effect sizes were calculated using the partial η^2^. Significant interaction results were further analyzed using paired *t* tests, and their effect sizes were calculated using Cohen *d*_*x*_.

## Results

### *Experiments 1 and 2*

#### *Pain Thresholds*

Average and standard deviation of CPTs were 13.86 ± 3.50°C (Experiment 1) and 13.34 ± 4.96 (Experiment 2), whereas HPTs were 42.62 ± 2.03 (Experiment 1) and 40.91 ± 2.11 (Experiment 2). Furthermore, the cold and warm temperatures at the TGT were 19.28 ± 2.81 and 40.72 ± 2.81 (Experiment 1) and 19.41 ± 2.20 and 40.59 ± 2.20 (Experiment 2).

#### *Temperature Matching*

**Figure 2:**
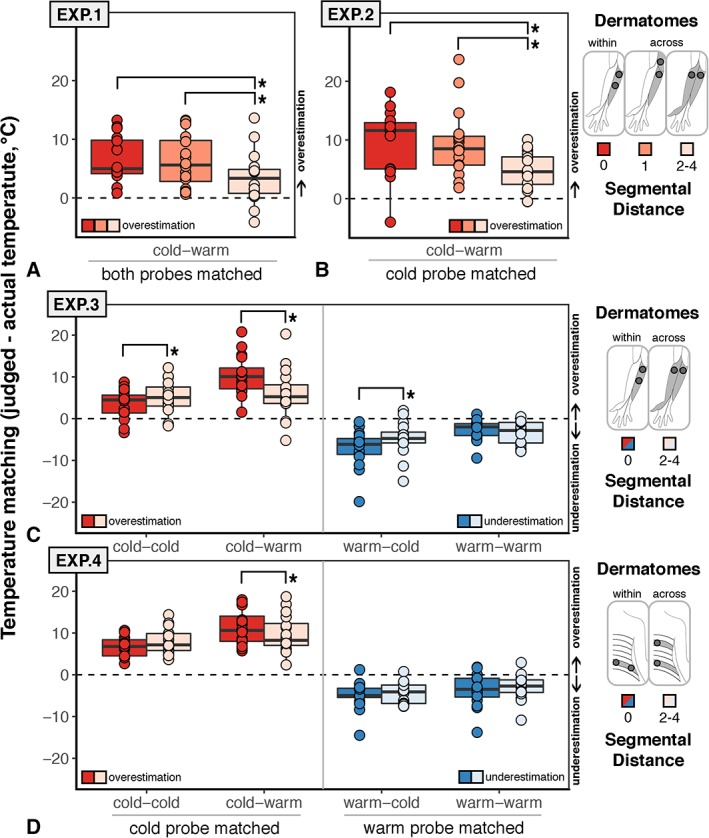
Results. Boxplots show the difference between judged and actual temperature at the matching tasks. (A) In Experiment 1, participants received thermal grill illusion (TGI; ie, cold–warm) stimulation and matched the overall temperature sensation from both thermodes. (B) In Experiment 2, participants received TGI (ie, cold–warm) stimulation and matched the sensation from the cold thermode. In both Experiments 1 and 2, participants systematically overestimated the overall or cold temperature across all conditions; however, in both experiments, overestimation was significantly reduced (ie, closer to the veridical temperature) when the segmental distance spanned 2 nonadjacent segments. (C, D) In Experiments 3 and 4, participants received TGI and non‐TGI stimulation consisting of 4 different temperature combinations (cold–cold, cold–warm, warm–cold, warm–warm) either on the forearm (Experiment 3, C) or on the lower back (Experiment 4, D). They matched either the cold thermode (cold–cold and cold–warm combinations) or the warm thermode (warm–cold and warm–warm combinations). In both Experiments 3 and 4, participants systematically overestimated the cold temperature; however, overestimation was larger for TGI versus non‐TGI combinations when the stimuli were applied within the same dermatome. No significant difference was found between TGI and non‐TGI combinations when the segmental distance spanned 2 nonadjacent spinal segments. Asterisks indicate statistical significance (p < 0.05) for the comparisons within vs across‐dermatomes. For simplicity, statistical significance is not depicted for comparisons across temperature combinations. [Color figure can be viewed at http://www.annalsofneurology.org]

Figure 2 shows boxplots, as well as single‐subject matched temperatures, as a function of the dermatomes stimulated and the corresponding spinal segments. The dependent variable is expressed as the difference between the perceived (matched) and the actual temperature on the skin. In Experiment 1, the dependent variable corresponded to the difference between the perceived temperature and the average temperature of the 2 thermodes. In contrast, in Experiment 2, the dependent variable corresponded to the difference between the perceived temperature and the temperature of the cold thermode. A positive difference always indicates that the temperature of the target stimulation felt warmer than it truly was (ie, temperature overestimation).

In line with the TGI phenomenon, overall participants overestimated the veridical average temperature (see Fig 2A) and the temperature of the cold stimulation (see Fig 2B). Crucially, we found that temperature overestimation depended significantly on whether cold and warm stimuli were delivered within the same dermatome or across dermatomes corresponding to adjacent or nonadjacent spinal segments in both Experiment 1 (*F*_2, 30_ = 4.95, *p* = 0.01, partial η^2^ = 0.25) and Experiment 2 (*F*_2, 30_ = 10.83, *p* < 0.001, partial η^2^ = 0.42). In both experiments, we found that temperature overestimation was similarly strong if cold and warm afferent signals were most likely located within the same spinal segment or across adjacent segments. In striking contrast, both these conditions differed from the critical condition, in which cold and warm afferent signals most likely spanned 2 or more segments. These results demonstrate that the strength of the illusion, as indicated by temperature overestimation, is modulated by the segmental distance between cold and warm afferents.

#### *Qualitative Perception*

At the postexperiment debriefing, participants were asked to select adjectives to describe the quality of their experience from a list of descriptors. They reported that the dominant thermal sensation was hot (Experiment 1: n = 7; Experiment 2: n = 8), warm‐to‐hot (Experiment 1: n = 6; Experiment 2: n = 5), cold (Experiment 1: n = 2; Experiment 2: n = 2), or equally cold and hot (Experiment 1: n = 1; Experiment 2: n = 1). Furthermore, participants also reported that the thermal sensation was burning (Experiment 1: n = 9; Experiment 2: n = 12), tingling (Experiment 1: n = 9; Experiment 2: n = 7), stinging (Experiment 1: n = 2; Experiment 2: n = 4), or pricking (Experiment 1: n = 2; Experiment 2: n = 8).

### *Experiments 3 and 4*

#### *Pain Thresholds*

Average and standard deviation of CPTs were 8.73 ± 4.93°C (Experiment 3, forearm) and 7.41 ± 3.74°C (Experiment 4, lower back), whereas HPTs were 43.99 ± 3.27°C (Experiment 3, forearm) and 43.62 ± 2.92°C (Experiment 4, lower back). Furthermore, the cold and warm temperatures at the TGT were 18.80 ± 3.18°C and 41.20 ± 3.18°C (Experiment 3, forearm) and 18.04 ± 3.81°C and 41.96 ± 3.81°C (Experiment 4, lower back).

#### *Temperature Matching*

Figure 2 shows boxplots, as well as single‐subject matched temperatures, as a function of temperature pairs (cold–cold, cold–warm, warm–cold, warm–warm), and dermatomal boundaries (within vs across) for Experiments 3 and 4. The dependent variable is expressed as the difference between the perceived (matched) and the actual temperature of either the cold or the warm thermode. Temperature overestimation (positive difference) was largely observed when participants were asked to match the cold thermode. Conversely, temperature underestimation (negative difference) was largely observed when participants were asked to match the warm thermode. In the statistical analyses, the sign of warmth underestimation was reversed to compare the magnitude of cold misperception versus warm misperception. However, we kept the original sign (negative difference) in Figure 2 to represent the overall positive difference when matching cold thermode and the overall negative difference when matching warm thermode.

In Experiment 3, the interaction between the 3 factors of interests (temperature 1 × temperature 2 × segmental distance) was significant (*F*_1, 15_ = 20.29, *p* < 0.001, partial η^2^ = 0.57; see Fig 2). In Experiment 4, the interaction between the 4 factors of interests (temperature 1 × temperature 2 × segmental distance × skin distance) was not significant (*F*_1, 15_ = 1.39, *p* = 0.26, partial η^2^ = 0.08). However, the 3‐way interaction (temperature 1 × temperature 2 × segmental distance) was significant (*F*_1, 15_ = 5.02, *p* = 0.04, partial η^2^ = 0.25). More specifically, in Experiments 3 and 4, we replicated the segmental distance effect. In line with Experiments 1 and 2, we showed that cold overestimation of TGI stimuli varied with segmental distance in both the forearm (cold–warm, 0 vs 2–4 segments, *t*_15_ = 2.90, *p* = 0.01, Cohen *d*_*x*_ = 0.72) and the lower back (cold–warm, 0 vs 2–4 segments, *t*_15_ = 2.41, *p* = 0.03, Cohen *d*_*x*_ = 0.60). This TGI overestimation effect was greater than unimodal spatial summation of cold when the stimuli were presented within dermatomes on the arm (0 segments, cold–warm vs cold–cold, *t*_15_ = 5.46, *p* < 0.001, Cohen *d*_*x*_ = 1.46) or lower back (0 segments, cold–warm vs cold–cold, *t*_15_ = 5.85, *p* < 0.001, Cohen *d*_*x*_ = 1.36). In contrast, the TGI overestimation effect was not significantly greater than unimodal spatial summation of cold when the stimuli were presented across dermatomes on the arm (2–4 segments, cold–warm vs cold–cold, *t*_15_ = 0.80, *p* = 0.44, Cohen *d*_*x*_ = 0.20) or lower back (2–4 segments, cold–warm vs cold–cold, *t*_15_ = 1.90, *p* = 0.08, Cohen *d*_*x*_ = 0.47).

The TGI overestimation effect was also greater than unimodal spatial summation of warmth when stimuli were presented within dermatomes on the arm (0 segments, cold–warm vs warm–warm, *t*_15_ = 5.44, *p* < 0.001, Cohen *d*_*x*_ = 1.36) or lower back (0 segments, cold–warm vs warm–warm, *t*_15_ = 5.24, *p* < 0.001, Cohen *d*_*x*_ = 1.31; see Fig 2). This effect was not found when stimuli were delivered across dermatomes on the arm (2–4 segments, cold–warm vs warm–warm, *t*_15_ = 1.56, *p* = 0.14, Cohen *d*_*x*_ = 0.39), but remained when stimuli were presented across dermatomes on the lower back (2–4 segments, cold–warm vs warm–warm, *t*_15_ = 4.33, *p* < 0.001, Cohen *d*_*x*_ = 1.08).

Warm underestimation was greater within dermatomes compared to across dermatomes on the arm (warm–cold, zero vs 2–4 segments, *t*_15_ = 3.08, *p* < 0.01, Cohen *d*_*x*_ = 0.77; see Fig 2C) but not on the back (warm–cold, zero vs 2–4 segments, *t*_15_ = 0.56, *p* = 0.58, Cohen *d*_*x*_ = 0.14; see Fig 2D). This result is consistent with the perception of burning cold from TGI stimulation, which is consistently reported in a lower percentage of cases compared to burning hot sensations. The observation that warm stimulation may be more often misperceived as intense cold in the within‐dermatome versus across‐dermatomes condition is in line with increased TGI integration at short segmental distances.

Finally, unimodal cold was perceived closer to the veridical temperature when stimuli were delivered within rather than across dermatomes on the arm (cold–cold, zero vs 2–4 segments, *t*_15_ = −2.48, *p* = 0.03, Cohen *d*_*x*_ = −0.62; see Fig 2). However, this segmental cold effect did not exceed the threshold of *p* < 0.05 on the lower back (cold–cold, zero vs 2–4 segments, *t*_15_ = −2.12, *p* = 0.05, Cohen *d*_*x*_ = −0.53). Furthermore, unimodal warm stimulation was perceived similarly within and across dermatomes both on the arm (warm–warm, zero vs 2–4 segments, *t*_15_ = −1.30 *p* = 0.22, Cohen *d*_*x*_ = −0.33) and lower back (warm–warm, zero vs 2–4 segments, *t*_15_ = 0.92, *p* = 0.37, Cohen *d*_*x*_ = 0.23). In summary, we replicated the TGI segmental distance effect across 4 different experiments and within 2 body parts, and demonstrated that this effect could not be explained by unimodal spatial summation of cold or warmth within and across dermatomes. Instead, we showed that the segmental effect was specific to TGI integration.

### *Meta‐Analytic Effect Size*

**Figure 3:**
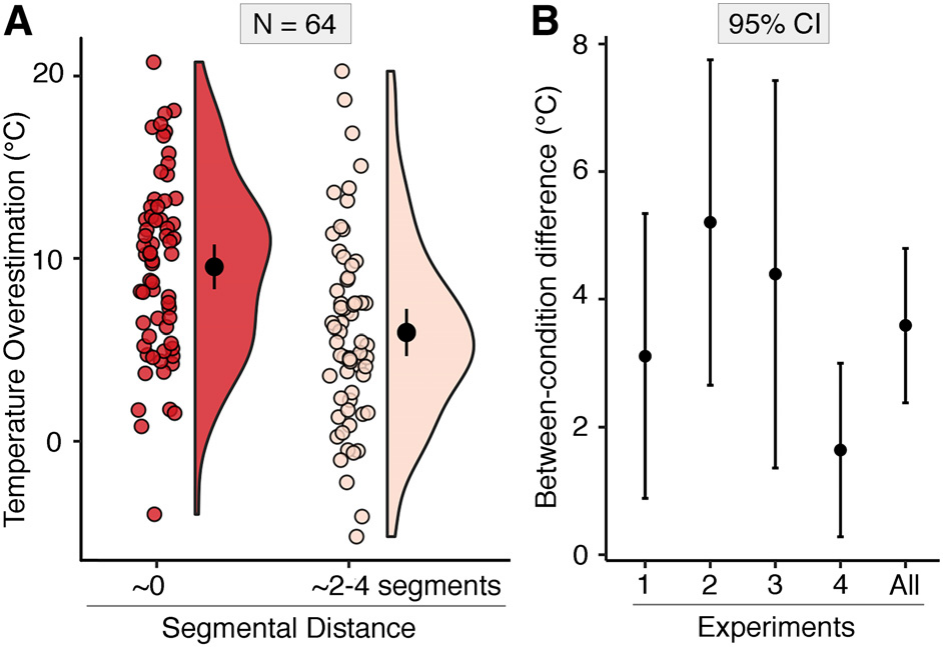
Meta‐analytic effect size. (A) Raincloud plot of the pooled data across experiments (N = 64) for the 2 key segmental conditions: short segmental distance (∼0 spinal segments) and long segmental distance (∼2–4 spinal segments). Each single‐subject value corresponds to the difference between judged and actual temperature at the matching tasks, separately for the minimal and maximal segmental distance conditions. Positive values correspond to cold overestimation, whereas negative values correspond to cold underestimation. The half violin plots depict the probability density of the data at different values and contain 95% confidence intervals (CIs) of the mean for the 2 conditions. (B) Ninety‐five percent CIs of the difference between the 2 key segmental conditions. Positive values correspond to a larger thermal grill illusion (TGI) effect for short versus long segmental distance. The differential TGI segmental effect is plotted for each experiment (n = 16), as well as the pooled data across experiments (N = 64). [Color figure can be viewed at http://www.annalsofneurology.org]

To summarize the spinal integration effect size across experiments, we calculated the magnitude of the TGI segmental effect, by subtracting the 0 segments effect (ie, increased temperature overestimation within spinal segments) from the 2 to 4 segments effect (ie, decreased temperature overestimation across nonadjacent spinal segments). Figure 3A depicts single‐subject values, confidence intervals, and the probability density of the data at different values, separately for the 2 key segmental conditions. In Figure 2B, we report the 95% mean confidence intervals of this TGI segmental effect for each experiment separately, as well as for the pooled data across experiments.

## Discussion

In 4 experiments, we showed that a cardinal feature of the TGI—misperception of cold—is influenced by low‐level integration mechanisms in the spinal cord. The strength of the TGI, as indexed by the degree of temperature overestimation, depended upon the segmental distance between cold and warm afferents. Overestimation of TGI stimuli was greater when cold and warm afferents were at a short distance within the spinal cord (ie, within the same segment or across adjacent segments), but was drastically reduced when cold and warm afferents corresponded to nonadjacent spinal segments. This effect was replicated in 2 different body regions (ie, forearm and lower back), and could not be explained simply by task demands or unimodal spatial summation.

The overestimation effect from TGI was best captured by asking participants to match the temperature of the cold thermode. Misperception of cold, rather than warm, is the primary feature of the TGI.^7^, ^11^, ^23^ We initially showed that the segmental distance effect was significant when matching the overall stimulus temperature (Experiment 1). However, the segmental effect was larger when participants matched the objectively cold thermode (Experiment 2). As the task instructions in these 2 experiments required participants to attend to either both thermodes simultaneously or only 1 thermode at a time, the difference in effect size between these conditions is likely due to spatial attention, which can dynamically modulate temperature and pain perception.^21^, ^22^

Crucially, TGI overestimation within dermatomes was significantly larger than the overestimation of unimodal cold stimuli, confirming a specific within‐segment TGI effect (Experiments 3 and 4). In contrast, overestimation from TGI and unimodal cold stimuli was highly similar when the constituent sensory signals projected to nonadjacent segments in the spinal cord. Furthermore, spatial summation of unimodal warmth was similar irrespective of spinal adjacency (Experiments 3 to 4). This pattern of results is in agreement with previous literature, indicating a lack of segmental modulation for unimodal warm and heat pain across 1,^24^ 2,^25^, ^26^ or more spinal segments.^27^, ^28^ These TGI and unimodal results were confirmed not only on the forearm, but also on the lower back, where the dermatomes are more clearly defined (Experiment 4). In summary, these results suggest that the TGI is abolished when cold and warm afferents are located >2 segments apart in the spinal cord. In contrast to this segmental TGI effect, we showed that unimodal spatial summation of warmth and cold is largely determined by spatial proximity on the skin, with minor dependence from segmental organization. These effects appear compatible with the hypothesis that temperature integration underlying the TGI is mediated by Lissauer tract neurons, which are known to form short‐range intersegmental connections across 1 or 2 spinal segments.^12^, ^13^

Our findings are seemingly inconsistent with a previous study on the spatial boundaries of the TGI, showing similar effects within and across dermatomes.^9^ However, in our experiments, we crucially compared 2 across‐dermatomes conditions based on segmental adjacency. Our manipulation enabled us to test the extent of spinal integration across spinal segments that are more or less interconnected via the Lissauer tract. This points to the fundamental contribution of spinal summation mechanisms in modulating the strength of the TGI; however, it does not rule out the contribution of other peripheral or supraspinal mechanisms. Peripheral spatial summation might be critical when stimuli are delivered within a small skin area, without any separation between thermodes.^24^, ^29^, ^30^ To limit such peripheral summation, we induced TGI by using only 2 stimuli that were individually adjusted to target specific classes of thermoreceptors, separated by at least 5cm. Conversely, cortical mechanisms might contribute to the TGI in the case of multisensory integration. For example, in previous studies the illusion was found to be modulated by interactions between thermal and proprioceptive inputs.^19^, ^31^ To control for thermal–proprioceptive interactions, we applied TGI stimulation on a static arm and carefully controlled spatial distance between stimuli.

In line with our spinal interpretation, previous functional magnetic resonance imaging studies found that thalamic activity is increased in response to TGI, but not to the constituent innocuous warm and cold temperatures.^32^ Similarly, a right insular region was shown to be active in response to paradoxical heat stimuli, but not dynamic cooling of the skin.^33^ These results imply that TGI integration can occur before the temperature‐related neural signals reach thalamic and insular regions. However, thermosensory perception is enabled by an extensive network of frontoparietal regions, indicating that supraspinal interactions are also fundamental for the generation of thermosensory perceptual experiences in both the innocuous^34^ and noxious range.^35^

Although our results are consistent with the view that integration of cold and warm afferents evoking TGI occurs at least partially at the spinal level, it remains unclear how different neurophysiological components, such as peripheral receptors, fibers, and crosstalk excitation or inhibition, contribute to heat and pain illusions. Previous work indicated several alternative neurophysiological mechanisms and pathways, including the difference in activity between cold‐specific and polymodal nociceptive neurons at the thalamocortical level,^7^, ^11^ summation by wide dynamic range neurons in the deep dorsal horn^4^, ^6^ and type 2 C‐afferents.^36^ More specifically, animal work suggests reciprocal cross‐inhibition between cold and warm afferents in the spinal cord.^37^, ^38^ Future work is needed to identify how the putative neurophysiological mechanisms interact with segmental‐based integration of cold and warm afferents in humans.

Our work is in agreement with recent empirical investigations demonstrating that touch, temperature, and pain signals interact in spinal microcircuits in the superficial dorsal horn,^37^ for example, through crosstalk between heat and cold circuits. This evidence for cross‐modal interaction and convergence at the spinal level is incompatible with the classic specificity theory (ie, of labeled lines), and is driving a paradigm shift in the way somatosensation is currently viewed. The emerging “population coding model” suggests that temperature and pain perception arises from the joint activity across several neuronal populations, rather than depending on neural signals of specific labeled lines, with 1 dominant line associated with each specific sensory quality.^39^, ^40^, ^41^, ^42^ Our experiments are consistent with the view that TGI perception is largely influenced by the pooled activity of spinal neurons within 1 or 2 spinal segments.

As a marker of this spinal interaction, the TGI might be an interesting tool to understand the disruption of temperature and pain perception in clinical populations, such as in the case of neuropathic pain of central origin. Surprisingly, the TGI has been used in only a few previous clinical investigations in neurological^43^, ^44^, ^45^ and psychiatric patients.^46^, ^47^, ^48^ Furthermore, although it is not part of the standard somatosensory assessment, it is qualitatively similar to the misperception of heat from dynamic cooling (ie, paradoxical heat sensations^15^), which is routinely tested in quantitative sensory testing^49^ and is a cardinal feature of several peripheral and central neuropathies.^50^ The TGI has been also considered qualitatively similar to cold allodynia,^7^ another symptom commonly reported in neuropathic pain patients corresponding to the perception of pain from mild cooling. Further research is required to investigate whether the method presented here can be used to further characterize the mechanisms underlying these different temperature and pain misperception symptoms and to noninvasively assess spinal integration functionality in clinical populations.

In summary, here we provide a novel noninvasive method to assess thermosensory integration in the spinal cord, as well as compelling evidence supporting the involvement of spinal mechanisms in modulating the perception of the TGI. In the future, this method can be adapted to test how spinal processing contributes to different somatosensory functions, across different parts of the body, and may ultimately enrich common neurophysiological clinical assessments.

## Author Contributions

F.F. conceived the study; all authors designed the study; F.F. acquired and analyzed data, and drafted figures; all authors wrote the paper.

## Potential Conflicts of Interest

Nothing to report.
